# Incidence of adverse reaction of drugs used in COVID-19 management: a retrospective, observational study

**DOI:** 10.1186/s40545-021-00370-3

**Published:** 2021-10-25

**Authors:** Jia Yin Lee, Abby Shoon Yeun Ang, Nurdalila Mohd Ali, Li Min Ang, Azura Omar

**Affiliations:** Department of Pharmacy, Sungai Buloh Hospital, Ministry of Health Malaysia, Sungai Buloh, Selangor Malaysia

**Keywords:** Adverse drug reactions, Clinical pharmacy, Drug safety, Drug monitoring, Pharmaceutical care

## Abstract

**Background:**

An urgent need for coronavirus infectious disease (COVID-19) treatment has resulted in off-label drug use. Although previous studies had investigated the adverse drug reaction (ADR) of the medications for COVID-19 in their respective local settings, the safety profile in a Malaysian setting remains unknown. Our study aims to establish the incidence of ADR for drugs used in COVID-19 management in a Malaysian tertiary hospital.

**Methods:**

This retrospective observational study enrolled patients started on drugs for COVID-19 in Sungai Buloh Hospital from 1 March 2020 to 31 May 2020. The clinical staging of COVID-19 patients was decided by the treating physician in accordance with the Clinical Management of Confirmed COVID-19 Case in Adults (Annex 2E). Suspected ADRs were evaluated with a trigger tool of pre-defined laboratory values or the adverse events listed in the registered product insert. Causality assessment was conducted when an ADR was suspected using the World Health Organization-Uppsala Monitoring Centre (WHO-UMC) system, and only cases classified as certain, probable and possible ADR were considered. Data analysis was completed with descriptive, univariate and multivariate analysis.

**Results:**

The study (*N* = 1,080) identified 217 patients (20.1%) who experienced ADR, with 246 adverse events detected. Most events were related to the gastrointestinal (43.5%), hepatobiliary (36.2%) and cardiac (16.3%) systems. The most commonly suspected drugs were atazanavir (52.7%), chloroquine (36.8%) and lopinavir/ritonavir (34.6%). The independent risk factors of ADR were female (adjusted odds ratio (OR): 1.53; 95% CI 1.06–2.20; *P* = 0.024), diagnosis of COVID-19 stage 3 (adjusted OR: 2.58; 95% CI 1.20–5.55; *P* = 0.015) and stage 4 (adjusted OR: 4.17; 95% CI 1.79–9.73; *P* = 0.001), and the number of COVID-19 drugs (adjusted OR: 3.34; 95% CI 2.51–4.44; *P* < 0.001). Only 49 adverse events (19.9%) were manually reported by healthcare professionals, with hyperbilirubinaemia (65.3%) and QT prolongation (28.6%) most frequently reported.

**Conclusion:**

Medications used in COVID-19 management had resulted in one in five patients experiencing ADR. Our study has provided an overview on incidence of ADR for off-label use of medications used in COVID-19 management, which suggests a similar safety profile when used for FDA-approved indications.

## Introduction

The World Health Organization (WHO) declared the novel coronavirus disease 2019 (COVID-19) to be an international public health crisis on 30 January 2020 [1]. Drugs that were previously used to treat severe acute respiratory syndrome (SARS) and Middle-East respiratory syndrome (MERS), such as chloroquine, hydroxychloroquine and interferon beta-1b, are now potential candidates to treat COVID-19. The rationale behind repurposing these agents is mostly based on the similar genetic proximity that these viruses share, resulting in an off-label use of these medicines [2].

Numerous clinical studies are currently being conducted globally to investigate the effectiveness of potential drug therapies for COVID-19. Although the current focus is mainly investigating the efficacy of drugs for COVID-19 treatment, priority should also be given to ensure medication safety due to a 44% increase in risk associated with off-label drug use [3].

Despite adverse drug reaction (ADR) being documented with registered indications, the safety profile of repurposed COVID-19 medications remains largely unknown with limited evidence. A multicentre, observational study on chloroquine use in COVID-19 patients (*N* = 197) concluded that well-established side effects occurred in 26.9% of chloroquine group, i.e. dizziness (10.2%) and nausea (9.1%) [4]. Meanwhile, a French study by Gerard et al. [5] found that among 120 cardiac events due to drugs for COVID-19 management (hydroxychloroquine, chloroquine, lopinavir–ritonavir and azithromycin), 36.6% of cardiac events were associated with serious QT prolongation of QTc value > 500 ms. An active monitoring study in China with COVID-19 patients on treatment (*N* = 217) recorded an ADR rate of 37.8%, with the main complaints including gastrointestinal disorders (23%) and liver system disorders (13.8%) [6]. In view of the scarcity of ADR reports from drugs for COVID-19 management, pharmacovigilance represents an important role in safety surveillance activity.

The National Pharmaceutical Regulatory Agency (NPRA) of Malaysia encourages ADR monitoring and reporting to determine the incidence of ADR, to identify predisposing risk factors for ADR and to maintain a national ADR database [7]. Previous studies have shown that ADR contributing factors include age, gender, multiple drug therapy, current disease, ethnic, genetic differences and pharmaceutical factors [8].

Reporting of suspected or known ADR of drugs when used in real healthcare settings can help to identify patterns and trends not found in clinical trials, resulting in heightened regulatory scrutiny and drug withdrawal based on risk–benefit ratio [9]. A systematic review by Hazell et al. [10] found that the reporting rate of ADR had been underwhelming, with a median of 94% underreporting rate (interquartile range 82–98%) after reviewing spontaneous reports from hospitals and general practices across 37 studies from 12 countries. Contributions from these reports are necessary to provide timely information about the safety of these experimental COVID-19 drug therapies and to promote rational drug use.

This study aims to establish the incidence of ADR due to off-label drugs used in COVID-19 management in a local tertiary hospital setting. Secondary outcome measures are to establish the type of ADRs, to identify potential risk factors for ADR and to evaluate the reporting rate of ADR for healthcare professionals.

## Methods

### Study design and population

A retrospective, observational study using electronic medical records (EMRs) from Sungai Buloh Hospital, the main referral hospital for COVID-19 in Malaysia, was conducted to include data from 1 March 2020 to 31 May 2020.

The diagnosis and clinical staging of COVID-19 patients were done in accordance with the local guideline, Clinical Management of Confirmed COVID-19 Case in Adult (Annex 2E) by the Ministry of Health, Malaysia [11]. Clinical staging was updated according to patients’ disease progression upon daily review (Table 1), with the highest clinical staging taken into account in this study. Patients started on medications for COVID-19 were included in the study. Children aged 12 years and below with COVID-19 were only started with symptomatic treatment, hence excluded from the study.

**Table 1:** Clinical staging of syndrome associated with COVID-19 [11]

Clinical stage	Disease progression
1	Asymptomatic
2	Symptomatic, no pneumonia
3	Symptomatic, pneumonia
4	Symptomatic, pneumonia, requiring supplemental oxygen
5	Critically ill with multi-organ involvement

Adapted from Ministry of Health, Malaysia

All patients with suspected or confirmed COVID-19 requiring off-label drugs were identified through a name list generated from the EMR and screened for ADR. Medications that were used in COVID-19 patients during the study period were chloroquine, hydroxychloroquine, lopinavir/ritonavir, atazanavir/ritonavir, ribavirin, interferon beta-1b, tocilizumab or favipiravir.

This study was registered with the National Medical Research Register and was approved by the institutional review boards of the Medical Research Ethics Committee of the Ministry of Health, Malaysia (NMRR-20-1120-55048 (IIR)).

### Active monitoring by investigators

A trigger tool (Table 2) consisting of laboratory parameters was prepared as per the definitions from previous guidelines or journals. Meanwhile, adverse events that cannot be defined by laboratory values were matched with adverse events listed in the registered product inserts to identify ADR.

**Table 2:** Trigger tool for ADR detection

Trigger items	Explanation
Exceeds 500 ms or there has been an increase of ≥ 60 ms compared with the pre-drug baseline value [12]	Drug-induced QT prolongation
Alanine aminotransferase (ALT) ≥ 5× or alkaline phosphatase (ALP) ≥ 2× and total bilirubin (TBL) ≥ 2× upper limit of normal (ULN) [13]	Drug-induced liver injury
Baseline < 180 umol/L: 50% increment from baseline or minimum increment by 40 umol/L; baseline > 180 umol/L: Minimum increment by 90 umol/L. Note: Drug exposure must be 24 h preceding the event [14]	Drug-induced acute kidney injury
< 50 × 10^9^ platelets/L [15]	Thrombocytopenia
< 1.5 × 10^9^ neutrophils/L [16]	Neutropenia
> 6.8 mg/dL (404 µmol/L) [17]	Hyperuricaemia
< 10 g/dL haemoglobin [18]	Drug-induced anaemia

Retrospective chart review was conducted by the investigators trained in pharmacovigilance, covering medical records on discharge summary, procedure notes, progress notes and laboratory data. Actions to minimise misclassification bias include screening medical records in a standardised manner and counterchecking detected ADR by another team member.

### Data collection

Patients’ characteristics (diagnosis, sex, age, one or more comorbidities and history of drug allergy), medications for COVID-19 and laboratory results, were documented with a case report form. History of drug allergy can be retrieved from the EMR in the form of free text entry in the progress note. Evaluation of ADR was done when the trigger item or adverse event was detected, with the suspected causative drug, time of onset, treatment of ADR and the clinical outcome being recorded.

### Causality assessment

The causality assessment of all suspected ADR was completed using the World Health Organization-Uppsala Monitoring Centre (WHO-UMC) system [19]. The relationship between the detected ADR and drugs was categorised as certain, probable, possible, unlikely, unclassified/conditional, or unassessable/unclassifiable, and only ADR cases categorised as certain, probable and possible were further analysed.

The seriousness of the ADR was ranked using the International Conference on Harmonization (ICH) E2A guideline (ICH E2A Clinical Safety Data Management: Definitions and Standards for Expedited Reporting). As defined by the guideline, a serious event or reaction is any untoward medical occurrence that at any dose: “(1) resulted in death; (2) is life-threatening; (3) required hospitalisation or resulted in prolongation of existing hospitalisation; (4) resulted in persistent or significant disability/incapacity, or (5) caused congenital anomaly/birth defect or medically important event or reaction that required medical/surgical intervention to prevent serious outcome” [20].

The clinical outcome indicators of ADR were recovered fully, recovering, not recovered, unknown, or fatal. The ADR was considered clinically cured with the disappearance of symptoms or recovery of the laboratory indexes to normal values. Patients were followed until recovery or discharge, whichever earlier, to determine the clinical outcome.

### ADR reporting by healthcare providers

A separate manual reporting of ADR was completed by the doctors and pharmacists in the facility. These ADR reports were documented in the case bank and submitted to NPRA, and matched with the ADR cases identified in the study to determine the rate of ADR reporting.

$$\mathrm{Reporting\, rate}=\frac{\mathrm{Number \,of\, ADR \,cases\, reported \,by \,healthcare \,providers}}{\mathrm{Total \,number\, of \,ADR\, cases \,identified\, in\, the\, study}}\times 100\%$$

### Data analysis

Data collected were analysed using the statistical software Statistical Package for the Social Sciences (SPSS) Version 24.0. All performed analyses were set as two-tailed with a confidence interval of 95% and *P* value less than 0.05 was considered statistically significant. Demographic data were analysed using descriptive analysis (frequency and percentage). The association of potential risk factors for ADRs was evaluated using both univariate, i.e. Chi-square test, Mann–Whitney test and simple logistic regression, as well as multivariate analysis with multiple linear regression.

## Results

A total of 1080 patients were included in this study. The characteristics of the participants were summarised in Table 3. There were more ADR cases in older patients and those with comorbidity (both *P* < 0.001). There were no significant differences in proportions of sex and history of drug allergy between the two groups.

**Table 3:** Demographics and clinical background of participants (*N* = 1080)

Variable	Patients with ADR (*n* = 217)	Patients without ADR (*n* = 863)	*P* value
**Age** (mean ± SD in years)	47.96 ± 16.50	40.07 ± 16.51	*<* 0.001^†^
**Sex**			
Male	137 (63.1%)	593 (68.7%)	0.116^‡^
Female	80 (36.9%)	270 (31.3%)	
**History of drug allergy**			
Yes	5 (2.3%)	26 (3.0%)	0.576^‡^
No	212 (97.7%)	837 (97.0%)	
**Presence of comorbidity**			
Yes	117 (53.9%)	294 (34.1%)	*<* 0.001^‡^
No	100 (46.1%)	569 (65.9%)	
**COVID-19 category**			
Patient under investigation	9 (4.1%)	80 (9.3%)	
Stage 1	1 (0.5%)	35 (4.1%)	
Stage 2	27 (12.4%)	357 (41.4%)	
Stage 3	85 (39.1%)	311 (36.0%)	
Stage 4	79 (36.4%)	58 (6.7%)	
Stage 5	16 (7.4%)	22 (2.5%)	
**Number of COVID-19 drug(s)**			
1	51 (23.5%)	704 (81.6%)	
2	96 (44.2%)	115 (13.3%)	
3	46 (21.2%)	30 (3.5%)	
4 and 5	24 (11.1%)	14 (1.6%)	

Data are *n* (%) or mean (± SD)

^†^Analysed with Mann–Whitney *U* test

^‡^Analysed with Chi-square (*X*^2^) test

### Classifications of ADRs

The adverse events were categorised according to system organ class (SOC) using WHO-Adverse Reaction Terminology (WHO-ART), as listed in Table 4. The most common ADRs detected were gastrointestinal disorders (43.5%), followed by hepatobiliary disorders (36.1%) and cardiac disorders (16.2%).

**Table 4:** Classification of ADR (*n* = 246)

ADR according to SOC	Incidence of ADR	Time to onset		
		Acute (within 60 min)	Subacute (1 to 24 h)	Latent (after 2 days)
**Blood and lymphatic system disorders**				
Anaemia	2 (0.8%)	0	1	1
**Cardiac disorders**				
QT prolongation	34 (13.8%)	0	9	25
Bradycardia	4 (1.6%)	0	1	3
ST elevation	1 (0.4%)	0	0	1
Palpitation	1 (0.4%)	0	0	1
**Gastrointestinal disorders**				
Diarrhoea	76 (30.9%)	0	49	27
Nausea and vomiting	27 (11.0%)	1	7	19
Abdominal pain	4 (1.6%)	0	2	2
**Hepatobiliary disorders**				
Hyperbilirubinaemia (without jaundice)	77 (31.3%)	0	17	60
Elevated liver transaminases	6 (2.4%)	0	0	6
Hyperbilirubinaemia (with jaundice)	5 (2.0%)	0	3	2
Elevated alkaline phosphatase	1 (0.4%)	0	0	1
**Nervous system disorders**				
Giddiness	3 (1.2%)	1	0	2
Headache	1 (0.4%)	0	1	0
**Renal and urinary disorders**				
Acute kidney injury	2 (0.8%)	0	1	1
**Skin and subcutaneous tissue disorders**				
Rash	2 (0.8%)	0	1	1

Incidence of ADR is reported as *n* (%), where % is calculated by number of ADR/*n*

### Drugs used in COVID-19 and ADR

Almost all patients (98%) were started on hydroxychloroquine. Nevertheless, ADRs were most commonly detected in atazanavir (52.7%), followed by chloroquine (36.8%) and lopinavir/ritonavir (34.6%), as seen in Table 5. The ADRs detected were classified as probable or possible. Most patients fully recovered from the effects of ADR, while the rest showed signs of recovering prior to discharge. No serious ADR was identified from all cases.

**Table 5:** Suspected drug, ADR, outcome and causality

Suspected drugs	Patient on treatment	ADR	Outcome		Causality^a^	
			Recovered	Recovering	Probable	Possible
Atazanavir	167	88 (52.7%)	57	29	57	29
Chloroquine	19	7 (36.8%)	7	0	0	7
Lopinavir/ritonavir	214	78 (36.4%)	75	3	18	60
Ribavirin	7	1 (14.3%)	1	0	1	0
Hydroxychloroquine	1035	118 (11.4%)	107	11	22	96
Interferon beta-1b	91	3 (4.4%)	3	0	0	3
Tocilizumab	8	0	0	0	0	0
Favipiravir	6	0	0	0	0	0

ADR is reported as *n* (%), where % is calculated by taking ADR/patient on treatment

^a^No ADR cases were classified as certain

Among 217 patients who experienced ADR, drug-related adverse reactions were highest (44.2%) in those started on two COVID-19 drugs (Table 3). The pairs of suspected drug with the highest rate of ADR were atazanavir–hydroxychloroquine (25.3%) and lopinavir/ritonavir–hydroxychloroquine (15.7%) (Table 6). The most common ADRs among these pairs were hyperbilirubinaemia (6.5%) and diarrhoea (8.3%), respectively.

**Table 6:** Incidence of ADR in different drug-pair combinations

COVID-19 drug pair	ADR
Atazanavir–hydroxychloroquine	55 (25.3%)
Lopinavir/ritonavir–hydroxychloroquine	34 (15.7%)
Lopinavir/ritonavir–interferon beta-1b	5 (2.3%)
Atazanavir–favipiravir	1 (0.5%)
Lopinavir/ritonavir–chloroquine	1 (0.5%)

ADR is reported as *n* (%), where % is calculated by taking ADR/patient with ADR

When used individually, the most common ADR detected was hyperbilirubinaemia (37.8%) for atazanavir and diarrhoea (21.2%) for lopinavir/ritonavir. Diarrhoea (25.4%) and QT prolongation (14.3%) were the most frequently detected ADR for hydroxychloroquine.

### Comorbidities and ADR

The incidence of ADR was higher in patients with one or more comorbidities (*P* < 0.001). Significant difference in incidences of ADR were detected in patients with chronic kidney disease (50%), cardiovascular diseases (39.7%), hypertension (30.5%) and diabetes mellitus (29.9%) (*P* < 0.05), as compared to those without these comorbidities (Table 7).

**Table 7:** Comorbidity and ADR

Comorbidity	Number of patients	Patients with ADR	*P* value^#^
Cancer	4	3 (75.0%)	0.006
Obesity	7	5 (71.4%)	0.001
Chronic kidney disease	38	19 (50.0%)	*<* 0.001
Cardiovascular diseases	58	23 (39.7%)	*<* 0.001
Hypertension	249	75 (30.1%)	*<* 0.001
Diabetes mellitus	157	46 (29.3%)	0.002
Chronic obstructive lung disease	4	1 (25.0%)	0.814
Asthma	47	10 (21.3%)	0.862

Patients with ADR are reported as *n* (%), where % is calculated by patients with ADR/number of patients

^#^Analysed with Chi-square (*X*^2^) test

### Risk factors of ADR

Using univariate analysis, factors that were associated with the occurrence of ADR include age, presence of comorbidity, patients diagnosed with COVID-19 stage 3 to 5 and number of COVID-19 drugs (Table 8). With every increment in age, the odds of experiencing ADR increases by 3% (crude odds ratio [OR] = 1.03, *P* < 0.05).

**Table 8:** Univariate and multivariate model of variables associated with ADR

Variable	Crude OR (95% CI)	*P* value	Adjusted OR (95% CI)^a^	*P* value
**Demographics**				
Age (mean ± SD in years)	1.03 (1.02, 1.04)	*<* 0.001	–	–
Sex				
Male	1	–	1	–
Female	1.28 (0.94, 1.75)	0.117	1.53 (1.06, 2.20)	0.024
**Clinical background**				
History of drug allergy				
Yes	0.76 (0.29, 2.00)	0.577	–	–
No	1	–	1	–
Presence of comorbidity				
Yes	2.26 (1.68, 3.06)	*<* 0.001	–	–
No	1	–	1	–
COVID-19 category				
Patient under investigation	1	–	1	–
Stage 1	0.25 (0.03, 2.08)	0.202	–	–
Stage 2	0.67 (0.30, 1.48)	0.319	–	–
Stage 3	2.45 (1.18, 5.07)	0.016	2.58 (1.20, 5.55)	0.015
Stage 4	12.11 (5.62, 26.10)	*<* 0.001	4.17 (1.79, 9.73)	0.001
Stage 5	6.47 (2.52, 16.60)	*<* 0.001	–	–
Number of COVID-19 drug(s)	3.94 (3.20, 4.85)	*<* 0.001	3.34 (2.51, 4.44)	*<* 0.001

^a^Forward and backward LR applied. Hosmer and Lemeshow = 0.121. Classification table = 83.2. No multicollinearity was detected. ROC = 0.83 (0.79, 0.86; *P* value ≤ 0.001). Only variables with *P* value < 0.05 were mentioned in the AOR

In multivariate analysis, the independent risk factors for the occurrence of ADR were female (adjusted OR: 1.53; 95% CI 1.06–2.20; *P* = 0.02), patients diagnosed with COVID-19 stage 3 (adjusted OR: 2.58; 95% CI 1.20–5.55; *P* = 0.015) and stage 4 (adjusted OR: 4.17; 95% CI 1.79–9.73; *P* = 0.001) and number of COVID-19 drugs (adjusted OR: 3.34; 95% CI 2.51–4.44; *P* < 0.001). Age, presence of comorbidity and diagnosis of COVID-19 stage 5 were not independent risk factors when analysed with multivariate analysis.

### Reporting rate of ADR by healthcare professionals

Among 246 ADR cases detected within the sample, only 49 cases (19.9%) were manually reported, in which 48 cases of the ADR reports were submitted by pharmacists, and one case was submitted by doctor. The most commonly reported ADRs were hyperbilirubinaemia (65.3%) and QT prolongation (28.6%), with the main suspected drug being atazanavir and hydroxychloroquine, respectively. There were no ADR reports received for gastrointestinal symptoms, despite it contributing to the most number of ADR cases detected.

## Discussion

Around 20% of hospitalised patients experienced ADR while receiving treatment for COVID-19 based on the findings of this study. In China, Sun et al. [6] reported a higher ADR incidence rate of 37.8% among their COVID-19 patients. A systematic review of 29 studies had published that the prevalence of ADR among inpatients (non-COVID-19 related) ranged from 1.6% to 41.4% [21]. In summary, ADR appears to be an event that occurs with drug use irrespective of indication. The predominant ADRs detected in this study were gastrointestinal and liver-related disorders, in agreement with the results by Sun et al. [6].

The ADRs detected in this study were classified as probable or possible. In practice, few reactions are considered as “certain” due to the presence of confounding factors in most cases, including the underlying diseases or concomitant drugs, that has to be considered during the evaluation of a causal relationship between a drug and an ADR [19, 22]. Meanwhile, the causality category “probable” indicates that the ADR is “unlikely to be attributed to the disease or other drugs”, whereas “possible” suggests that the ADR “could be explained by the disease or other drugs” [19]. No serious ADR was detected from this study, possibly due to timely discontinuation of medication by the treating physicians.

Risk factors for ADR determined in this study using univariate analysis include age, presence of comorbidity, diagnosis of COVID-19 stage 3 to 5, and the number of COVID-19 drugs. The independent risk factors of ADR identified with multivariate analysis were female, diagnosis of COVID-19 stage 3 and stage 4, and the number of COVID-19 drugs.

It has been established that the elderly population is more prone to developing ADRs [23]. Our study has shown that the risk of ADR increases by 3% with each increasing year in age. The older patients could be affected due to the presence of chronic disease, polypharmacy and age-related physiological changes that may alter the drug’s pharmacokinetics and pharmacodynamics [24]. On a related note, patients with comorbidity are found to have a 2.26-fold risk of experiencing ADR compared to patients with no known medical illnesses, possibly due to the complex interactions between multiple drugs and disease states [25].

Several studies had identified the female gender as one of the potential risk factors of experiencing ADR [23, 25, 26]. Our study reported a 1.53-fold risk in females to experience ADR than males, which corresponds to current literature [23, 25, 26]. Postulated factors such as differences in circulating hormonal levels, having more prescription drugs, and a higher dose used in relation to their body weight were used to explain this phenomenon [26].

The recommended treatment from Guidelines on COVID-19 Management in Malaysia (as of 24 March 2020), at the point of data collection, was hydroxychloroquine in patients with COVID-19 stage 2, with the addition of protease inhibitors (atazanavir or lopinavir/ritonavir) in stage 3 (with warning signs) as well as stage 4 [27].

Patients who developed ADR were mostly diagnosed with COVID-19 stage 3 (39.3%, adjusted OR: 2.62; 95% CI 1.22–5.63; *P* = 0.014) or stage 4 (36.1%, adjusted OR: 4.16; 95% CI 1.78–9.71; *P* = 0.001). The use of these protease inhibitors could have contributed to most of the ADRs observed in this group of patients. Hyperbilirubinaemia is a well-documented ADR of atazanavir, whereas diarrhoea is a common ADR for lopinavir/ritonavir [28]. This study demonstrated that off-label use of medications in COVID-19 patients showed similar safety profile when used as per FDA-approved indications [29–31].

Multivariate analysis revealed that the risk of ADR was increased by 3.34 times with every addition of one COVID-19 drug to the treatment regime. Despite numerous studies investigating the effect of the number of drugs on ADR occurrence, the mechanism remains unestablished. It was proposed that drug–drug interactions could have contributed to most ADR, which arose from pharmacokinetic or pharmacodynamic interactions [8, 23]. Examples of pharmacokinetic interactions include induction or inhibition of metabolising enzymes and displacement of a drug from plasma protein binding sites; on the other hand, pharmacodynamic interactions involve additive or antagonistic pharmacological effects [8].

ADR reporting is defined as identify, quantify, evaluate and prevent risk related to the use of marketed drugs [32]. All healthcare workers are responsible for documenting and reporting clinically important ADR to the national pharmacovigilance centre [33]. However, voluntary reporting may lead to substantial underreporting of ADR [33]. During the study period, our facility’s reporting rate was 19.9%, which was translated to an underreporting rate of 80.1%. A systematic review investigated the extent of underreporting of an ADR to spontaneous reporting systems in hospitals and general practices across 37 studies from 12 countries and found that the median underreporting rate was 94% (interquartile range 82–98%) [10].

Common reasons for underreporting among healthcare workers were inexperience (reporting only serious and unexpected ADR), insecurity (notification made only when damage is certain), indifference (postponing notification of damage due to various excuses) and lack of training in pharmacovigilance (unfamiliar with procedures to report ADR) [34]. These factors are consistent with the observations made in our facility, as only severe ADR reports (e.g. hyperbilirubinaemia and QT prolongation) were received.

Almost all (98%) of ADR reports were submitted by the pharmacists during the study period. This corresponds to the local nationwide ADR reporting pattern, where majority of the ADR reports (68.1%) were submitted by pharmacists and only 16.5% were submitted by doctors in 2018 [35]. A similar trend was observed where pharmacists were the highest notifiers for ADR cases in other parts of the world, implicating that pharmacists may have greater engagement in pharmacovigilance issues [22]. Hence, continuous education for other healthcare professionals is much needed to encourage adherence to ADR surveillance [22, 34].

This study provided an overview on the incidence of ADR for the off-label use of medications among COVID-19 patients during the early days of the pandemic in a Malaysian COVID-19 referral hospital. It was worth noting that the WHO clinical management interim guidance (as of 13 March 2020) states “there is no current evidence to recommend any specific anti-COVID-19 treatment for patients with confirmed COVID-19”, and advises that “investigational anti-COVID-19 therapeutics should be used only in approved, randomised, controlled trials” [36].

The latest WHO Therapeutics and COVID-19: Living Guideline (released 31 March 2021) recommends against administering hydroxychloroquine, chloroquine and lopinavir/ritonavir for the treatment of COVID-19 due to a lack of evidence in improving patient outcome, coupled with the risk of increased adverse events [37]. However, our findings suggest a similar safety profile when prescribed for FDA-approved indications. Only favipiravir, interferon beta-1b and tocilizumab are currently in use in our facility.

There are some limitations to this study. As patients were enrolled for retrospective chart review using convenience sampling, this may result in skewed patient demographics. Although measures to reduce misclassification bias were implemented, it may be impossible to completely eliminate this error. The causal relationship between the drug and ADR may be influenced by other confounding factors, especially in nonspecific complaints (eg. gastrointestinal symptoms), despite only taking into account related adverse events that occurred after drug use. A lack in a comparator group also makes it difficult to rule out the interference caused by COVID-19 infection.

The interpretation of the study may also be limited by the minimal usage of favipiravir and tocilizumab during the study period, resulting in poor representation of ADR cases for these medications. Thus, further investigations may be necessary to determine the rate of ADR associated with favipiravir and tocilizumab.

## Conclusion

The repurposed medications for COVID-19 management suggest a similar safety profile when compared to the usage of medications prescribed for FDA-approved indications. This present study found that 20.1% of patients experienced ADR from off-label drugs used in COVID-19 management. The ADRs detected mostly affect the gastrointestinal, hepatobiliary and cardiovascular systems. Female, diagnosis of COVID-19 stage 3 and stage 4, and the number of COVID-19 drugs were identified as independent risk factors of ADR. Regardless, low reporting rate among healthcare professionals remains an issue to be tackled to provide a more complete and accurate representation of ADR cases. Given the uncertainty surrounding COVID-19, more research is warranted in ADR surveillance to maximise patient safety.

## Data Availability

The datasets used and/or analysed during the current study are available from the corresponding author on reasonable request.

## Abbreviations

ADR: Adverse drug reaction
ALP: Alkaline phosphatase
ALT: Alanine aminotransferase
COVID-19: Coronavirus disease 2019
EMR: Electronic medical record
FDA: Food and Drug Administration
ICH: International Conference on Harmonization
MERS: Middle-East respiratory syndrome
NPRA: National Pharmaceutical Regulatory Agency
OR: Odds ratio
SARS: Severe acute respiratory syndrome
SOC: System organ class
SPSS: Statistical Package for the Social Sciences
TBL: Total bilirubin
ULN: Upper limit of normal
WHO: World Health Organization
WHO-ART: World Health Organization-Adverse Reaction Terminology
WHO-UMC: World Health Organization-Uppsala Monitoring Center
